# Supplementary material for: Optimal control methods for nonlinear parameter estimation in biophysical neuron models
Source: PLoS Comput Biol. 2022 Sep 15;18(9):e1010479. doi: 10.1371/journal.pcbi.1010479 (PMC9514669; doi:10.1371/journal.pcbi.1010479)
Supplement: Attachment — Submitted filename: Kadakia_PloSCompBiol_review.pdf [file pcbi.1010479.s001.pdf]

Review of Optimal control methods for nonlinear parameter estimation in biophysical neuron Models by Kadakia.

The paper develops computational optimization to estimate the state and parameters of dynamical systems. The Lorenz model and the Hodgkin-Huxley model are used as examples of application. The paper begins by observing that a nudging term added to the equations of motion of the observed state variables biases the model towards synchronization to the data. The magnitude of this term is initially chosen empirically. The author then proposes the Pontryagin minimization scheme to obtain an optimal value for the nudging strength. The tradeoff is a cost function (Eq.11) that now only depends of state variables and momenta at the expense of additional constraints.

The development of efficient methods for estimating the parameters of biological models is highly topical and of critical importance for gaining a first principle understanding of intracellular processes. The method reported here makes progress in this direction.

The introduction fails to justify the motivation for the present approach. In what way does this approach improve over the state of the art nonlinear optimization? It would help to have a summary of the key results in the introduction and how these improve over to the main parameter estimation methods described in the literature. Lines 2-46 could better focus on the challenges and shortcomings of other approaches rather than recalling conductance models which are well-known

The author presumably considers well-posed inference problems where the data are generated by the same exact model used to infer the parameters. The author will presumably intend the present method for inferring parameters from recordings of actual neurons. One would anticipate that setting the U parameter according to Eq.9 will block synchronization of the model to the data in the case of ill-posed problems. Could the author comment of this and any contingency?

The Lorenz model has 2 observed variables whereas the HH model has only one. A minimum number of observations is generally needed for all Lyapunov exponents of the system to be negative. In the cases considered here it is unlikely the number of observations will be sufficient for this to occur. Is a control variable such as Tikhonov regularization needed for the parameter search to converge? What is the effect of fixing U (eq.9) on the stability of convergence?

More information on the computational implementation of the method would be welcome. For example, how are the constraints in Eq.10 discretized over the observation window?

The paper as written in the present version will resonate with a limited audience of specialists. To reach a broader audience a critical discussion of the advantages and drawbacks should be included in necessary with figures of merit. The elliptic style and use of different words for describing the same thing can be unnecessarily confusing. For example: **l.52** “Coupling” is between what and what? **l.97** “Covariance matrix” what matrix are you referring to? **Fig.1A** what is the horizontal axis? **Eqs 8->11** switch from continuous to discrete time.

In summary, I believe the paper meets the criteria of originality and potential importance to the neuroscience community. However, the claims are not well situated with respect to the existing literature. The advantages (and drawbacks) of the method for inferring information from biological neurons should be extensively covered if the paper is seen to be relevant to the neuroscience community.
