# Supplementary material for: Optimal control methods for nonlinear parameter estimation in biophysical neuron models
Source: PLoS Comput Biol. 2022 Sep 15;18(9):e1010479. doi: 10.1371/journal.pcbi.1010479 (PMC9514669; doi:10.1371/journal.pcbi.1010479)
Supplement: Attachment — Submitted filename: response_to_reviewers_1.pdf [file pcbi.1010479.s002.pdf]

Reviewer #1:
The paper develops computational optimization to estimate the state and parameters of dynamical systems. The Lorenz model and the Hodgkin-Huxley model are used as examples of application. The paper begins by observing that a nudging term added to the equations of motion of the observed state variables biases the model towards synchronization to the data. The magnitude of this term is initially chosen empirically. The author then proposes the Pontryagin minimization scheme to obtain an optimal value for the nudging strength. The tradeoff is a cost function (Eq.11) that now only depends of state variables and momenta at the expense of additional constraints.

The development of efficient methods for estimating the parameters of biological models is highly topical and of critical importance for gaining a first principle understanding of intracellular processes. The method reported here makes progress in this direction. The introduction fails to justify the motivation for the present approach. In what way does this approach improve over the state of the art nonlinear optimization? It would help to have a summary of the key results in the introduction and how these improve over to the main parameter estimation methods described in the literature. Lines 2-46 could better focus on the challenges and shortcomings of other approaches rather than recalling conductance models which are well-known.

I thank the reviewer for the remarks and suggestions. This is a very fair point. I have reduced the discussion of conductance-based models in the Introduction, and added more explicit explanation of the limitations of filtering methods for parameter estimation. In addition, I have added a summarizing paragraph in the Introduction specifically noting the advancements of this technique over other approaches for parameter estimation.

Moreover, in the Discussion, I have added an extra paragraph (lines 427-439) explicitly noting the advantages of OC-DSPE over related optimization-based techniques. I have also expanded the discussion of tradeoffs between sequential and optimization-based approaches for inference in neural systems (lines 446-469). Finally, I have added **new Table 1** in the Discussion comparing OC-DSPE to other related techniques, as a guide for the reader. This table includes advantages and drawbacks that will situate the work better in context.

The author presumably considers well-posed inference problems where the data are generated by the same exact model used to infer the parameters. The author will presumably intend the present method for inferring parameters from recordings of actual neurons. One would anticipate that setting the U parameter according to Eq.9 will block synchronization of the model to the data in the case of ill-posed problems. Could the author comment of this and any contingency?

The Lorenz model has 2 observed variables whereas the HH model has only one. A minimum number of observations is generally needed for all Lyapunov exponents of the system to be negative. In the cases considered here it is unlikely the number of observations will be sufficient for this to occur. Is a control variable such as Tikhonov regularization needed for the parameter search to converge? What is the effect of fixing U (eq.9) on the stability of convergence?

I comment on both of these 2 questions here, since they are related.

In the case of model misspecification, it is indeed the case that the estimates will eventually degrade. As a test of this, I have added **new Fig. 7** in which I have attempted to fit the model to observations from a related but neuron model with additional ion channel. In this case, the mis-specified model can still be fit very well, producing predictions that still align with the true trajectory of the original neuron. OC-DSPE can thus handle model misspecifications (which is an important test, since all ion channels of real neurons may not be known), although it is the case that if the model is entirely wrong, the system parameters estimates will be off and the predictions will be poor.

As far as the effect on synchronization, I note that the effect of fixing *U* on both synchronization and the stability of convergence is not entirely interpretable – by Eq. 9, the optimally controlled *U* is a function of *p*, which is itself dynamic. In fact, once the system is converted to the estimation dynamics (Eq. 10), the system is purely Hamiltonian, so all negative Lyapunov exponents are always paired with positive ones. Unless the system is purely oscillatory, then dissipative modes coexist with expanding modes. This persistent stability is the reason that the dynamics cannot be integrated forward directly and are instead used as constraints which are to be satisfied within the optimization problem.

In this sense, it is perhaps not appropriate not to identify the synchronization or stability of the system with the convergence of the parameter search. Instead, the estimation dynamics provide a stringent set of constraints that define the optimally estimated trajectory, and can more effectively nudge the optimal estimate to the global minimum. This is in contrast to the usual synchronization approach, where the controlled system (with known parameters) is integrated forward and will synchronize if the observability is sufficient. It is possible that the state and parameters can be estimated accurately in the OC-DSPE optimization framework, without satisfying the minimum observability conditions or synchronization in the usual sense.

More information on the computational implementation of the method would be welcome. For example, how are the constraints in Eq.10 discretized over the observation window? The paper as written in the present version will resonate with a limited audience of specialists. To reach a broader audience a critical discussion of the advantages and drawbacks should be included in necessary with figures of merit. The elliptic style and use of different words for describing the same thing can be unnecessarily confusing. For example: l.52 “Coupling” is between what and what? l.97 “Covariance matrix” what matrix are you referring to? Fig.1A what is the horizontal axis? Eqs 8->11 switch from continuous to discrete time.

I have added **new Table 1** comparing several related approaches for state and parameter estimation in neural and dynamical systems to give the reader a better sense of how this technique is situated in the literature.

Note that the full details of the computational implementation are given in the Methods section – for example, the discretization scheme of Eq. 10 is Hermite-Simpson (lines 538-558). I have gone through the text and tried to tighten wording and be more explicit as needed, throughout.

In summary, I believe the paper meets the criteria of originality and potential importance to the neuroscience community. However, the claims are not well situated with respect to the existing literature. The advantages (and drawbacks) of the method for inferring information from biological neurons should be extensively covered if the paper is seen to be relevant to the neuroscience community.

Reviewer #2:
This paper introduces a new method for inferring the state and parameters of nonlinear stochastic dynamical systems where there are fewer measurements than the number of states. The method, termed OC-DSPE, combines a dynamic nudging approach with optimal control. By applying OC-DSPE to the chaotic Lorenz96 system and the Morris-Lecar neuron model, the author shows that the new method is superior to ordinary least squares and DSPE without the optimal-control component.

The paper is well written. The approach and result description, including figures, is clear. The method seems to offer a useful new approach to an important general problem in neuroscience. There are however several major issues regarding the method's applicability and advantages in the context of neural data analysis that are not adequately addressed. Additional analyses based on the points raised below are therefore crucial for appreciating the usefulness of the new approach in answering neuroscientific questions:

1. Noise: the paper's objective as stated in the abstract is joint state and parameter estimation in neural State Space Models (SSM). These are usually understood to refer to doubly stochastic dynamical systems. However, only measurement noise is considered, while neural recordings also often include process noise as described by the Langevin equation (Eq. 2). How does OC-DSPE fair in such scenario? Besides, only isotropic Gaussian measurement noise is considered, and its variance is assumed to be known. Can noise variance be also inferred as a model parameter? How about nondiagonal covariance matrices?

The reviewer raises a good point in terms of applicability to real-world data. It is indeed of interest to see how OC-DSPE fares in a situation in which the observations result from latent state space dynamics with non-zero process/Langevin noise. Thus, I have now generated a **new Figure 6** with synthetic data from the Morris-Lecar system, by integrating the model dynamics forward but adding a $\xi(t)$ noise term to the dV/dt equations – this term effectively acts as a noisy current input into the neuron. The standard deviation of $\xi(t)$ is chosen as varied between 10 and 100 (in the units of the ionic current terms and the injected current I). For comparison, I has dynamical range ~[0, 200].

With input noise at the level of $\sqrt{\langle\xi\xi\rangle} = 50$ pA, observations are now highly corrupted, so much so that in addition to significant changes in the subthreshold dynamics, entire spikes are mis-timed. Despite this, parameters are predicted sufficiently well, giving accurate predictions with very accurate subthreshold dynamics and spike timing. Even at the level of $\sqrt{\langle\xi\xi\rangle}$ =100 pA, spike timing is largely preserved, though there are changes in the spike shape and some missed spikes.

OC-DSPE as formulated does not directly estimate measurement noise variances, consistent with prior works on inference in neural systems (Nogaret et. al. *Sci. Rep*. 2016, Abarbanel et. al. *SIAM J. Appl. Dyn. Sys*. 2009, Taylor et. al. *PLoS Comp. Biol.* 2020, Huys & Paninski, *PloS Comp. Biol.* 2009, Ye et. al. *Phys. Rev. E*. 2015, Paninski et. al. *J. Comput. Neurosci.* 2008, Moye & Diekman, *J. Math. Neurosci.* 2018,). This is not typically seen as a limitation, since measurement noise is known from experimental setups. For example, in *in vivo* or *in vitro* measurements, the size of response fluctuations in response to zero or weak signal directly quantifies the measurement noise. I mention this limitation in the Discussion (lines 470-479).

2. Measurement: Each measurement here is assumed to equal one state plus noise. However, in in vivo recordings from, e.g., multielectrode arrays, measurements usually come from multi-unit activity. They are therefore (an approximately linear) mixture of several states. Can OC-DSPE handle such a situation and infer a mixing matrix as an unknown parameter, i.e., the output matrix in SSMs?

OC-DSPE, as formulated, does not directly infer measurement matrices, though this could in principle be treated by adding measurement matrices as an added parameter. The reviewer is right that this is a

limitation. As such, this work is at this stage primarily applicable for *in vitro* data. I mention this limitation in the Discussion (lines 470-479), and leave open the possibility of promoting measurement matrices to parameters and jointly estimating them alongside model parameters, as a potential for future studies.

3. Dynamics: The method assumes a full knowledge of the state dynamics. However, in neural recordings not all conductance types present in each neuron type are known a priori. What is OC-DSPE's solution to underdefined state equations?

Oftentimes, anatomical considerations can greatly constrain the type of channels present (Daou, Ross, et al, *J. Neurophys.* 2013), but there is always a level of uncertainty. On the one hand, the model might assume ion channels that are not present or present weakly (very small conductances); conversely, the model may be a simplification of the true dynamics. As with any state-space estimation technique, OC-DSPE is inherently limited by presumed knowledge of the dynamical model.

Still, to give some understanding of the limitations of OC-DSPE in the case of unknown channels, I have redone an estimation of the Morris-Lecar model, now using model generated from a model with an extra *persistent sodium* channel, modeled on neurons in a previous work (Daou, Ross, et al, *J. Neurophys*. 2013). The data was fit to a neuron model lacking this channel. The results, shown in (**new Fig. 7**), show that this extra channel can affect spike timing and can result in missed spikes, so it has measurable effects on the data. However, OC-DSPE is still able to fit the under-determined model to this data, with very accurate predictions. This results from a slight modification of some of the parameters. Thus, even with under-specified models, satisfactory model fits can be found for observed data, indicating that the inference procedure is robust to qualitative errors in the model. In sum, missing channels does not render the inference procedure of the *known* channels ineffective.

4. Predictions: One major advantage of SSMs is their ability to conduct in silico experiments and make predictions of future dynamics (for instance, how a neuron would behave under stimulation conditions that were not used during the inference stage). Are there advantages for using OC-DSPE in such analyses? That the method is more accurate suggests that it may make better predictions, but it would be useful to see that in practice.

Note that prediction is an inherent aspect of OC-DSPE: the optimally accurate estimate in Fig. 4 and 5 is determined by minimizing the error over the predicted portion (t = 100-200 ms), which is generated by integrating the model dynamics for a novel stimulus, using the estimated parameters. In all examples in Fig. 4 and 5, the stimulus for the predicted (future dynamics) portion is distinct from the portion used for estimation, so indeed OC-DSPE can give accurate predictions for novel stimuli.

In principle, OC-DSPE has no inherent advantage in the prediction aspect – estimation procedures producing more accurate parameter estimates will typically have better predictions. Still, to make the comparisons more explicit, I've added **new subfigure 4F-G,** showing predicted parameters and predicted traces for OC-DSPE, DSPE, and least squares, respectively, for two different measurement noise levels. This illustrates the superiority of OC-DSPE in the accuracy of predicted parameters, and this is reflected in more accurate predictions to novel current stimuli.

5. Comparisons: OC-DSPE was compared to simpler methods and was shown to perform better. However, the paper mentions other approaches that are similar in methodology (quasi-static variational assimilation and variational annealing). What are the advantages of OC-DSPE over this class of methods?

I have added **new Table 1** in the Discussion comparing several related approaches for state and parameter estimation in neural and dynamical systems to give the reader a better sense of how this

technique is situated in the literature. I have also discussed there the relationship between variational annealing and constrained least squares (which was used in the paper as a comparison). Indeed, as implemented in the paper, the constrained least squares approach is approximately equivalent to variational annealing.

Reviewer #3:
This paper proposes a new method, based on chaotic synchronization and optimal control theory, for inferring the parameters of conductance-based neuronal models directly from voltage traces. Results are presented demonstrating that the proposed method, OC-DSPE, yielded more accurate parameter estimates from synthetic data generated by the Lorenz96 system (when only 2 of the 10 state variables are observed) compared to 2 prior techniques. The OC-DSPE method is then used to estimate linear and nonlinear parameters from synthetic voltage traces generated by the Morris-Lecar neuronal model in response to a current pulse and a chaotic stimulating current.

Comments:

This paper is well-written and addresses an important challenge in computational neuroscience. While conductance-based models can be fit to data from voltage-clamp recordings on individual ionic currents, it is often the case that the only data available for model fitting are voltage traces from current-clamp recordings. This is a very difficult parameter estimation problem since only one of the model's many state variables is directly observed. Thus, an improved method for addressing this challenge would be a significant contribution to the field. However, some major changes to the manuscript are needed to demonstrate that the proposed method does indeed address this challenge.

Thanks to the reviewer for the encouraging comments and remarks.

1) The manuscript includes a direct comparison of the performance of the proposed OC-DPSE method to the prior methods of DSPE and least-squares minimization on data from the Lorenz96 system, but no such comparison is provided for data from the Morris-Lecar model. Since the Lorenz96 system is not a neuronal model, and the focus of the manuscript is on parameter estimation in biophysical neuron models, it would significantly strengthen the manuscript if a direct comparison of the performance for the proposed and prior techniques were provided for the Morris-Lecar model as well.

Absolutely a fair point. I have now added **new subfigure 4F-G**, which shows a comparison of the estimated Morris-Lecar model conductances against both DSPE and least squares, for two noise levels. All of the optimization parameters were the same between the 3 estimation techniques for an apples-to-apples comparison. OC-DSPE is clearly superior in both accuracy (the optimal estimate is accurate) and robustness (many runs find the optimal estimate), particularly as the noise increases. For example, for high noise, out of all 25 runs, neither DSPE nor least squares find estimates of g_{slow} that are near its true value of 20.0 (4th subplot in 4f). The degraded parameter estimates with DSPE and least squares are reflected in poorer predictions to novel stimuli (Fig. 4G).

2) The last paragraph in the "Optimally-controlled dynamical parameter inference" section of the Results (lines 202-220) does a nice job of relating the OC-DSPE method to previous approaches utilizing homotopy continuation and extensions of variational annealing to Hamiltonian manifolds. It ends by saying that Algorithm 1 is in much the same spirit as [49]. It would be helpful to add a concluding sentence here indicating what makes the proposed OC-DSPE method distinct from these previous approaches.

I have added a couple sentences (lines 227-229) summing this up. I have also added **new Table 1** in the Discussion, which characterizes many recent techniques for parameter estimation, and indicates how these different approaches differ in what they are able to estimate and how they carry out the estimation.

3) The manuscript emphasizes that the proposed method is tailored to situations with considerable measurement noise, however the type of noise used in the "Numerical experiments with the Morris-Lecar model", where uncorrelated Gaussian noise is added to the ground truth voltage, does not seem very biological to me as it doesn't appear to affect the timing of action potentials. Is this type of noise present

in in vitro current-clamp electrophysiology recordings? A more relevant type of noise, it seems to me, would be some form of dynamical noise (representing unmodeled synaptic inputs, or ion channel noise perhaps) that causes irregularities in the timing of action potentials. Would this type of noise still be considered measurement noise or instead some kind of model noise, and would OC-DSPE still perform well in the face of it? Even if results are not shown with this type of noise, a discussion of the characteristics of the noise encountered in actual experimental voltage traces and how they relate to the numerical experiments shown here and the way that noise is incorporated into the OC-DSPE framework is warranted.

Yes, that is a good point. The noise included is sort of measurement noise (say from the electrode). While this noise is clearly an experimental limitation that should be considered, it is not reflective of internal fluctuations, like signaling noise within the neuron itself. As the reviewer states, internal noise is a key consideration, because it affects timing of action potentials.

Thus, I have now generated a **new Figure 6** with synthetic data from the Morris-Lecar system, by integrating the model dynamics forward but adding a $\xi(t)$ noise term to the dV/dt equations – this term effectively acts as a noisy current input into the neuron. The standard deviation of $\xi(t)$ is chosen as varied between 10 and 100 (in the units of the ionic current terms and the injected current I). For comparison, I has dynamical range ~[0, 200].

With input noise at the level of $\sqrt{\langle\xi\xi\rangle} = 50$ pA, observations are now highly corrupted, so much so that in addition to significant changes in the subthreshold dynamics, entire spikes are mis-timed. Despite this, parameters are predicted sufficiently well, giving accurate predictions with very accurate subthreshold dynamics and spike timing. Even at the level of $\sqrt{\langle\xi\xi\rangle}$ =100 pA, spike timing is largely preserved, though there are changes in the spike shape and some missed spikes.

4) Does the proposed OC-DSPE method fall under the general umbrella of data assimilation methods? I'm asking because many of the references cited (11 of them by my count) contain the term data assimilation (or a variant of it) in their title, yet the word assimilation only appears once in the manuscript itself (on line 216). It might help orient readers with regards to the existing literature if it is explicitly stated whether OC-DSPE should be considered as a new variational data assimilation algorithm, or as something else.

Yes it does! I opted to avoid the term data assimilation (DA) as it is only used in some pockets of the state and parameter inference community. This is particularly true in the neuroscience community, where the terminology has been used liberally by certain labs, but is absent in others. Still, I agree with the reviewer that I should mention the connections and give some background on the terminology. I have added a paragraph in the Discussion summing this up (lines 459-469) and indeed noting that OC-DSPE can be viewed as a variational data assimilation approach.

5) I have two questions about lines 97-99 which reads "As will be discussed below, the more pressing issue is that sequential estimators such as both nudging and the Kalman filter are not directly suited for the estimation of static parameters."
First, is it appropriate to refer to nudging as a sequential estimator? I ask because in the Introduction, it seems that chaotic nudging synchronization is included in the paragraph that starts on line 38 as "An alternative to all of these sequential filtering approaches is the direct optimization of a posterior distribution over, jointly, the states at all timepoints and the unknown parameters."
This also leads to my second question, which is where is it discussed below that nudging and the Kalman filter are not directly suited for the estimation of static parameters? I found this confusing because line 170 says that DSPE is "built naturally on nudging synchronization" and earlier in the Introduction references are cited [5, 25, 26] that use a sequential filtering approach for parameter estimation. Perhaps,

rather than alluding to a discussion that will come later, it would be good to include a sentence or two about why nudging and the Kalman filter are not directly suited for the estimation of static parameters immediately following lines 98-99.

This was a confusing oversight on my part. Yes, nudging and filtering (any type of filtering) are both sequential estimators because they update estimates sequentially with each new observation, simply using the estimate from the previous time – as opposed to optimization techniques which treat all data simultaneously. So the current statement in the text is correct. However, it is unclear as the reviewer states. The reviewer suggests an excellent alternative in explaining this, which I've implemented in lines 101-105.

Minor comments:

1) Abstract, second sentence: replace "neurons models" with "neuron models"
2) Line 76: replace "an D-dimensional" with "a D-dimensional"
3) Line 170: replace "DSPE built" with "DSPE is built"
4) Equation 9: replace "observed)," with "observed)."
5) Line 185: replace "the the" with "the"
6) Line 250: replace "near on" with "near" or "on"
7) Line 294: extra space after "1" before the period
8) Line 315: replace "as" with "is"
9) Lines 376 and 379: the opening quotation mark is reversed in "nudged" and "noise-regulation"
10) Line 489: missing space between the comma and "and"

Thank you for the very close reading!
